# Death In-doors

**Published:** 1876-01

**Authors:** 


### DEATH IN-DOORS.

Multitudes of persons have a great horror of going out of doors for fear of taking cold; if it is a little damp, or a little windy, or little cold, they wait, and wait, and wait; meanwhile weeks and even months pass away, and they never, during that whole time, breathe a single breath of pure air. The result is, they become so enfeebled that their constitutions have no power of resistance; the least thing in the world gives them a cold; even going from one room to another, and before they know it they have a cold all the time, and this is nothing more or less than consumption. Whereas, if an opposite practice had been followed of going out for an hour or two every day, regardless of the weather, so it is not actually falling rain, a very different result would have taken place. The truth is, the more a person is out of doors, the less easily does he take cold. It is a widely known fact that persons who camp out every night, or sleep under a tree for weeks together, seldom take cold at all.

The truth is, many of our ailments, and those of a most fatal form, are taken in the house, and not out of doors; taken by removing parts of clothing too soon after coming into the house; or lying down on a bed or sofa when in a tired or exhausted condition from having engaged too vigorously in domestic employments. Many a pie has cost an industrious man a hundred dollars. A human life has many a time paid for an apple dumpling. When our wives get to work they become so interested in it that they find themselves in an utterly exhausted condition; their ambition to complete a thing, to do some work well, sustains them until its completion, and the moment it is completed the mental and physical condition is one of exhaustion, when a breath of air will give a cold, to settle in the joints to wake up next day with inflammatory rheumatism; or with a feeling of stiffness or soreness, as if they had been pounded in a bag; or a sore throat to worry and trouble them for months; or lung fever to put them in the grave in less than a week.

Our wives should work by the day, if they must work at all, and not by the job; it is more economical in the end to see how little work they can do in an hour, instead of how much. It is slow, steady, continuous labor which brings health and strength and a good digestion. Fitful labor is ruinous to all.

